# New Insights Into the Pharmacological Management of Postoperative Pain: A Narrative Review

**DOI:** 10.7759/cureus.23037

**Published:** 2022-03-10

**Authors:** Victor Mayoral Rojals, Moises Charaja, Oscar De Leon Casasola, Antonio Montero, Marco Antonio Narvaez Tamayo, Giustino Varrassi

**Affiliations:** 1 Pain Unit, Department of Anesthesiology, Hospital Universitari de Bellvitge (IDIBELL), L'Hospitalet de Llobregat, ESP; 2 Department of Anaesthesiology, Pediatric National Health Institute, Lima, PER; 3 Department of Anesthesiology, Buffalo School of Medicine, Buffalo, USA; 4 Department of Surgery, University of Lleida, Lleida, ESP; 5 Pain Medicine Unit, Hospital Obrero, La Paz, BOL; 6 President, Paolo Procacci Foundation, Rome, ITA

**Keywords:** preemptive analgesia, opioids, multimodal analgesia, postoperative pain, analgesia

## Abstract

Postoperative pain is prevalent and often undertreated. There is a risk that untreated or suboptimally treated postoperative pain may transition into chronic postoperative pain, which can be challenging to treat. Clinical guidelines recommend the use of multimodal analgesia, including non-steroidal anti-inflammatory drugs (NSAIDs), acetaminophen, and, in some cases, opioids. NSAIDs are a broad class of drugs with different attributes such as cyclo-oxygenase (COX)-1 or COX-2 selectivity, onset of action, and analgesic potency. NSAIDs are associated with gastrointestinal and cardiovascular side effects and should be administered at the lowest effective dose for the shortest effective duration but can be effective in postoperative pain. The role of opioids in postoperative analgesia is long-standing but has recently come under scrutiny. Opioids are often used in multimodal analgesic combinations in such a way as to minimize the total consumption of opioids without sacrificing analgesic benefit. Special clinical considerations are required for surgical patients already on opioid regimens or with opioid use disorder. A particularly useful fixed-dose combination product for postoperative analgesia is dexketoprofen-tramadol, which confers safe and effective postoperative pain control and reduces the risk of persistent postoperative pain.

## Introduction and background

Inadequately controlled postoperative pain continues to be a problem despite the fact that there is an armamentarium of pain relievers that can be deployed. Postoperative pain is associated with increased morbidity and dysfunction and can delay time to ambulation and prolong the hospital length of stay [1]. When acute postsurgical pain is not appropriately managed, it may develop into chronic postoperative pain, which, in turn, may lead to dysfunction, disability, and depression, and be difficult to manage [2]. Postoperative pain typically follows a relatively predictable trajectory, with the most intense pain occurring at first and dissipating as tissue heals [2]. Acute postoperative pain management involves pharmacologic treatments, which can sometimes be combined in multimodal analgesic regimens. Multimodal pain regimens use two or more agents with complementary mechanisms of action in such a way that overall opioid consumption is reduced without sacrificing analgesic benefit [3,4]. While opioids have long been a mainstay of acute postoperative pain management, there is increasing scrutiny on their use and efforts to reduce or even eliminate them in this setting. Multimodal analgesia and an increased understanding of acute pain following surgery can help reduce acute postoperative pain and possibly minimize its chance of chronification.

## Review

The roles of NSAIDs for postoperative pain

The clinical guidelines for the management of postoperative pain issued by the American Pain Society, the American Society of Regional Anesthesia and Pain Medicine, and the American Society of Anesthesiologists’ Committee on Regional Anesthesia makes a strong recommendation based on high-quality evidence that clinicians provide adult and pediatric patients paracetamol or acetaminophen (APAP) or nonsteroidal anti-inflammatory drugs (NSAIDs) as part of a multimodal analgesic regimen for the control of postoperative pain unless there are specific contraindications [5]. NSAIDs can have powerful central effects similar to those of opioids.

NSAIDs are a broad class of drugs [6]. All NSAIDs block cyclo-oxygenase (COX) enzymes and inhibit the local production of prostaglandins. NSAIDs may have a selective affinity for either the COX-1 or COX-2 enzyme (coxibs). Ibuprofen inhibits both COX-1 and COX-2 enzymes approximately equally [6]. In simple terms, NSAID-induced prostaglandin inhibition results in their anti-inflammatory effect and contributes to analgesia; however, multiple mechanisms are involved. For example, dexketoprofen trometamol decreases the transduction of the painful stimulus by inhibiting prostaglandin synthesis at the brain level while simultaneously decreasing nociceptive responses with a potency similar to that observed with opioid receptor agonists. Dexketoprofen enhances this antinociceptive effect by activating mechanisms linked to endogenous opioids and the serotonergic pathway [7].

Acute postoperative pain occurs with tissue damage that triggers the production of membrane phospholipids and phospholipidase A2 and launches the arachidonic acid cascade leading to the production of COX-1, COX-2, lipoxygenase, and downstream prostaglandin production [8]. Eicosanoids are a broad class of molecules derived from 20-carbon polyunsaturated fatty acids, such as, but not limited to, arachidonic acid. Among the many eicosanoids in the body are prostaglandins, thromboxanes, leukotrienes, and lipoxins. They are considered to be autocrine/paracrine hormones because they act on adjacent or nearby cells over short time periods. They regulate physiologic functions by inhibiting certain forms of synthesis and play essential roles in numerous cardiovascular and other physiologic functions [9]. Inhibition of prostanoid synthesis has long been an important drug target and led to the development of acetylsalicylic acid (ASA), acetaminophen (paracetamol), and NSAIDs. While all of these agents have anti-inflammatory, antipyretic, and analgesic properties, there are marked distinctions among the agents. For example, ASA has antipyretic but only limited anti-inflammatory effects. Guidance for using NSAIDs recommends prescribing the lowest effective dose for the shortest effective period of time, prescribing them to patients at a low risk of thrombotic events, and monitoring NSAID patients for increases in blood pressure, edema, renal dysfunction, and gastrointestinal (GI) symptoms such as bleeding [10].

When the analgesic efficacy of various NSAID agents was compared to their relative anti-inflammatory effects in a murine study using the paw edema test, differences emerged with ketoprofen appearing to be the most effective of the various agents [11]. A systematic review and meta-analysis likewise confirmed that oral ketoprofen was the most effective of the NSAIDs [12].

Rapid onset of analgesic action is an important prescribing consideration for treating acute postoperative pain. In a study validating the speed of onset of various NSAIDs in dental pain, dexketoprofen 25 mg was faster (~30 minutes) than diclofenac 50 mg (~60 minutes), tramadol 100 mg (~120 minutes), and piroxicam 20 mg (~120 minutes) [13]. The number needed to treat (NNT) for ≥ 50% pain relief of acute moderate-to-severe acute postoperative pain is 3.3 and 2.5 for ibuprofen 50 and 400 mg, respectively, versus 2.7 for diclofenac 50 mg [14]. In another meta-analysis for moderate-to-severe postoperative pain relief, the NNTs were 3.3 and 2.7 for ibuprofen 200 and 400 mg, respectively; diclofenac had an NNT of 2.3 and 1.8 for 50 and 100 mg, respectively [15].

Prescribing choices for any analgesic must consider safety and adverse events, which vary among agents. COX-1 inhibitors are more associated with GI side effects than coxibs, which are more associated with cardiovascular adverse events [16]. Other factors may increase a patient’s risk of GI adverse events with NSAIDs, including older age (risk ≥ 65 years and greater risk > 70 years), underlying severe illness, *Heliobacter pylori* infection, history of peptic ulcer, alcohol and/or tobacco use, and the use of more gastrolesive NSAID agents such as ketorolac or piroxicam. Patients taking two or more NSAIDs concurrently or an NSAID plus ASA, anti-platelet agents, anticoagulants, corticosteroids, and selective serotonin reuptake inhibitors (SSRIs) are also at an elevated risk of GI side effects [11].

The risk of rare side effects, such as hemorrhagic stroke, should also be taken into account. Taken as a drug class, NSAIDs conferred a slight, statistically insignificant risk of hemorrhagic stroke with a pooled risk ratio (RR) of 1.09, but when evaluated individually, the risk was greater for diclofenac and meloxicam with a RR of 1.27 for both; rofecoxib (since withdrawn from the market) had a RR of 1.35 [17]. A nested case-controlled study evaluated the risk of ischemic stroke with various NSAIDs (n=4,593,778 new NSAID users) and reported that ketorolac (odds ratio (OR) 1.46), traditional NSAIDs (OR 1.16), and coxibs (OR 1.08) were associated with a heightened risk of ischemic stroke. Ischemic stroke risk was greater in younger patients, males, and those with a history of ischemic stroke or transient ischemic attack [18]. It is not clear if concurrent use of anticoagulants, antiplatelets, or ASA might affect this risk.

NSAIDs have been associated with heart disease [19]. In a nested, case-controlled study using healthcare databases, 92,163 hospital admissions were determined among patients who started NSAID therapy at some time between 2000 and 2010; these were matched with 8,246,403 controls. The use of any NSAID within a 14-day window of hospital admission was associated with a 19% elevated risk of heart failure hospitalization (OR 1.19) compared to the use of any NSAID > 183 days in the past [19]. The lowest OR for heart failure admission was 1.16 for naproxen and the highest was 1.83 for ketorolac. The risk increased in some NSAIDs with high doses (defined as double or more the daily dose equivalent), and there was no evidence that celecoxib, in particular, increased the risk of heart failure hospitalization when used in therapeutic dose ranges [19].

All NSAIDs have been associated with an elevated risk of acute myocardial infarction (AMI), with the risk most pronounced in the first month of NSAID therapy. In a systematic review of a database cohort of 446,763 individuals, taking any dose of any NSAID for one week, one month, or over the course of a month increased the patient’s risk of AMI. The likelihood that specific NSAIDs are associated with an AMI range from OR 1.58 for rofecoxib to 1.53 for naproxen, 1.50 for diclofenac, 1.48 for ibuprofen, and 1.24 for celecoxib. Note that risks do not appear to increase for use >1 month compared to a one-month course, but higher doses of NSAIDs are associated with greater risk [20].

Liver damage may also occur with NSAIDs. In a case-controlled study (179 cases matched to 1,770 controls), the OR for acute serious liver injury associated with any NSAID was 1.69; nimesulide (OR 2.1) was associated with the greatest risk. Hepatotoxicity also occurred with ibuprofen both at therapeutic dose ranges and high doses (OR 1.92 and 3.73, respectively) and ketoprofen at doses ≥ 150 mg (OR 4.65) [21].

Kidney damage related to NSAIDs may relate to either increased blood pressure associated with COX-2 inhibition or decreased blood pressure associated with COX-1 inhibition. Renal dysfunction and acute kidney injury may occur with the use of any NSAID in a dose-dependent fashion. The risk is most pronounced in the elderly, those concurrently taking ACE inhibitors or angiotensin-II receptor blockers, and/or diuretics. Individuals with renal dysfunction should use NSAIDs only with great caution under close clinical supervision, if at all [22].

The use of NSAIDs for acute postoperative pain control deserves prudent clinical assessment. From a cardiovascular perspective, there is no “completely safe NSAID.” To the extent possible, patients with cardiovascular risk factors or a history of heart disease should avoid NSAID treatment. If it is determined by the prescriber and patient that the benefits of NSAID therapy outweigh the risks, then NSAIDs may be considered at the lowest effective dose and for the shortest possible period of time.

Opioids in postoperative pain management

Acute postoperative pain generally follows a well-defined trajectory with the most intense pain immediately following surgery, lessening day by day as the tissue heals. Postoperative pain is highly prevalent among surgical patients, with its duration and intensity varying by type of surgery. In a survey of 250 adults in the United States who had recently undergone surgery, 80% reported acute pain after surgery, and of this population, 86% described this pain as moderate-to-severe or very severe [23]. As hospital stays shorten and more surgery migrates to outpatient or ambulatory facilities, pain is often after discharge [23]. In day-surgery patients, a survey of 89 consecutive patients found that 40% reported moderate-to-severe pain in the first 24 hours after being released from the ambulatory surgery center and 25% needed to contact a healthcare provider after surgery for pain management [24]. Day surgery poses new challenges for safe and effective outpatient pain management [25].

Multimodal analgesia may be defined as pain treatment involving the use of two or more agents or nonpharmacological approaches with complementary mechanisms of action [3,4]. In acute pain associated with a peripheral trauma, such as postoperative pain, pain is perceived via peripheral nociceptors at the site. Localized peripheral pain may be treated with topical anesthetics, topical capsaicin, topical or oral NSAIDs, oral opioids, oral acetaminophen, oral anticonvulsants, or some combination. The pain site may be treated with nonpharmacological means, such as cryotherapy, heat therapy, continuous passive motion, or touch therapy. Pain signals traveling via the peripheral neural network to the dorsal root ganglion, the spinal cord, and then to the brain allow for pharmacological treatment along this pathway using opioids, local anesthetics, acetaminophen, anticonvulsants, α-2-adrenergic agonists (gabapentinoids), and N-methyl-D-aspartate receptor (NMDA) antagonists. Nonpharmacologic treatments to interrupt this pain pathway include transcutaneous electrical nerve stimulation (TENS), acupuncture, and massage [26].

An often overlooked aspect of postoperative pain care is patient education [5]. Prior to surgery, patients should be advised what sort of pain to expect and how to report pain levels, and be given a care plan so they understand how postoperative pain can be controlled. The appropriate route of administration is an important consideration: it must be acceptable and comfortable for the patient, appropriate for the agent and care, and ideally simple and clinically convenient [5].

The role of patient-controlled analgesia (PCA) with opioids after surgery may be effective in appropriate patients [27]. Appropriate candidates for opioid PCA are those who will require prolonged analgesia and have the cognitive ability to understand how the PCA device works and what its limitations are. PCA administration by proxy is generally not recommended except in rare situations. PCA is preferred over intermittent bolus dosing of IV opioids and is associated with better pain control and fewer side effects [5]. Opioids are increasingly coming under scrutiny because of concerns about tolerance, use disorder, and side effects, but short courses of opioids may be appropriate in selected patients for acute postoperative pain control [28].

Postoperative analgesia can be more challenging in patients already taking opioids before their surgery. This includes both chronic opioid therapy and opioid use disorder. The clinician should first determine the patient’s history of opioid use, current opioid regimen, and discuss postsurgical pain control strategies with the patient and family. Despite regular opioid use, such patients may still experience moderate-to-severe postoperative pain that is difficult to control. The clinician may wish to consider referral to a pain specialist for the use of certain nonpharmacologic approaches such as TENS, adjuvant treatments, peripheral regional analgesia, and the use of ketamine. For such patients, the role of PCA opioids is unclear. The clinical team should discuss postoperative opioid-tapering strategies with the patient before surgery and resume opioid use at the target doses after discharge [5].

While opioids are being scrutinized for postoperative analgesia, they are routinely administered perioperatively. The effects of specific anesthetics and analgesics, such as opioids, on natural killer (NK) cells are complex and possibly adverse [29]. The question has been posed as to whether or not opioid-free anesthesia (OFA) might be possible [30]. OFA is based on a paradigm that avoiding opioid anesthesia would reduce postoperative pain and improve outcomes, [31], but it has not been thoroughly studied [32,33]. OFA may be especially beneficial for certain patient populations, such as obese patients, patients with active opioid use disorder, high-risk patients, those with obstructive sleep apnea, chronic pain patients, and others [34].

It has been speculated that so-called “preemptive analgesia,” that is, opioids administered during the preoperative phase (prior to anesthesia), might decrease postoperative pain [35]. In a systematic review of 20 clinical studies (n=3,761) using various pre-incisional analgesic regimens, investigators could find no clinically important effect for preemptive analgesia on postoperative pain, but stopped short of saying there was no evidence for this lack of effect [36]. When a systematic analysis reviewed preoperative analgesic regimens compared to postoperative interventions via the same route in 66 studies (n=3,261), the evidence for preemptive opioid analgesia was equivocal [37]. In fact, an observational study of 123 fast-track total knee arthroscopy patients found that chronic preoperative use of opioids increased acute pain following surgery [38]. An observational study of 68 chronic pain patients scheduled for orthopedic surgery compared those taking opioids preoperatively to controls and found the opioid group (28/68) had higher scores on preoperative hyperalgesia tests, consumed significantly more morphine postoperatively (19.1 vs. 9.38 mg, p=0.001), had greater pain intensity in recovery, and had a higher cumulative consumption of postoperative morphine at 72 hours (39.8 vs. 25.6 mg, p=0.02) [39].

Preemptive opioid administration has been associated with hyperalgesic response. A murine study was designed to evaluate how ketamine might affect postoperative morphine analgesia on mice pretreated with sufentanil the day before surgery. Sufentanil administered one day before surgery triggered a hyperalgesic response to mechanical and thermal stimuli for up to four days. Ketamine treatment on the day before surgery enhanced the analgesic effect of morphine the day after surgery [40]. In a study of 21 healthy male volunteers, participants were administered either low-dose (1 µg/kg) or high-dose (10 µg/kg) IV fentanyl, and then cold pressor pain intensity levels and hyperalgesia were evaluated using intracutaneous electrical stimulation. High-dose IV fentanyl could be associated with better pain control at administration but was associated with greater pain sensitivity 4.5 to 6.5 hours thereafter (“hyperalgesia”) [41]. A study of 100 coronary artery bypass graft patients found that perioperative fentanyl was associated with postoperative tactile allodynia and thermal hyperalgesia in a dose-dependent fashion, with high-dose fentanyl (5-10 µg/kg/h) associated with a greater degree of allodynia and hyperalgesia than low-dose fentanyl (1-3 µg/kg/h). The decreased tactile and thermal pain thresholds returned to baseline values at about postoperative day 7 [42]. A systematic review of 27 randomized controlled trials (n=1,494) found that patients treated with higher intraoperative doses of opioids had greater postoperative pain intensity, consumed more morphine at 24 hours after surgery, but showed no significant differences in the rates of nausea, vomiting, or drowsiness. Most of the studies used in this analysis involved remifentanil [43]. Both opioid tolerance, a state of adaptation in which a drug loses its effect over time, and hyperalgesia, in which the patient has increased sensitivity to pain, may contribute to postoperative pain [44]. Proper selection of anesthetics is recommended to prevent postoperative pain exacerbation by way of opioid-induced hyperalgesia (OIH) [45].

The consumption of opioids after surgery varies widely among patients and may be related to a number of factors including patient preferences, genetics, environmental factors, patient history, type of surgery, cultural factors (whether or not pain is viewed as something to be endured or something to be avoided), and others [46].

Multimodal analgesia in the treatment of postoperative pain

The evidence-based approach to acute postoperative pain favors multimodal analgesia [47], which reduces side effects without sacrificing pain relief [48]. Poorly controlled acute postoperative pain may result in worse outcomes. Pain can elevate heart rate, even to the point of tachycardia, while simultaneously reducing blood flow, allowing for cardiac ischemia as the heart demands more oxygen than the body cannot deliver [49]. A total of 100 patients with or at high risk of coronary artery disease were monitored for one week following major non-cardiac surgery. Ischemia was defined as episodes of reversible ST-segment depression ≥ 1 mm or elevation ≥ 2 mm above baseline, adjusted for respiratory and positional variables [49]. In this study, 27% of patients experienced a total of 437 ischemic episodes in the first postoperative week with a total ischemic episode duration of 18,658 minutes, equal to a mean of 1.8 minutes per episode. Ischemia was most severe on postsurgical days 0 to 3, and 57% of ischemic episodes could be associated with tachycardia. Interestingly, 84% of these ischemic episodes were asymptomatic. Serious adverse events in postoperative patients occurred in five patients (unstable angina, myocardial infarction, cardiac death), and all were preceded by an ischemic episode occurring at least one day before [49].

Postoperative pain may also encourage shallow breathing, which, in turn, might lead to atelectasis, hypercarbia, and hypoxia, setting the stage for pneumonia [50]. Unrelieved postoperative pain can also delay time to ambulation, impede recovery, and prolong rehabilitation [51].

While acute postoperative pain is sometimes viewed in a limited way as a neurological-anatomical event, it is actually far more complex, involving multiple neural, hormonal, and emotional responses working in a peripheral-central nervous system feedback loop. Neural plasticity refers to the ability of the central nervous system to adjust itself both in structure and function to accommodate circumstances such as injury, pain, a changing environment, aging, or other factors [52]. The brain’s pain matrix involves the complicated interplay of multiple neuronal signaling cascades and bidirectional communications between the immune and nervous systems; chronic pain can occur when these functions become maladaptive [53].

While much has been written in the literature about central sensitization, peripheral sensitization must also be considered in the context of persistent postoperative pain. Peripheral sensitization involves hyper-responsiveness to stimulation and lowered thresholds of the nociceptive neurons in the periphery. Peripheral sensitization may occur following insult or injury to the tissue and/or with inflammation [54].

Neuromodulating substances influence synaptic plasticity, which appears to play a crucial role in the pathogenesis of pain following tissue injury [55]. Maladaptive synaptic plasticity in the brain and spinal cord involves central sensitization, the driving force behind chronic pain syndromes [56]. While microglia and other neuromodulators have been implicated in many slowly developing conditions, such as Alzheimer’s disease and other neurodegenerative diseases, their effect on neuronal and synaptic processes in the body in response to pain can occur in a matter of minutes [57]. While peripheral damage can cause mechanical allodynia in the periphery, it can also trigger microgliosis in the spinal cord, leading to morphological changes in the microglia and possibly resulting in chronic pain [58].

Opioids are paradoxically both analgesic and hyperalgesic. OIH lowers the patient’s pain threshold and thus increases the perception of pain intensity. In a survey of 850 clinicians who treated chronic pain patients, 76% reported that they treated patients with OIH in their practice and 38% stated that OIH affected >5% of their chronic pain patients. When it occurred, OIH was treated by these clinicians most frequently with a reduced opioid dose (68%), the addition of a nonopioid adjuvant agent (62%), or opioid discontinuation (48%) [59].

Gabapentinoids (pregabalin and gabapentin) used perioperatively have been shown in numerous clinical studies to reduce postoperative pain [60-62]. Gabapentinoids bind to the α-2Δ subunit of the P/Q type voltage-gated calcium channels and, in that way, reduce the release of glutamate [63]. This is thought to inhibit pain signal transmission and impede central sensitization. There is also evidence that gabapentinoids activate noradrenergic pathways in the brain and spinal cord to inhibit pain signals [64]. Although gabapentinoids are used all over the world, dosing is generally higher in the United States than in Latin America. Pregabalin 300 mg or gabapentin 1,200 mg two hours prior to surgery and then pregabalin 150 mg or gabapentin 600 mg postoperatively for one to 14 days are recommended, with adjustments for renally impaired patients [63]. Note that more studies have been conducted using the older agent, gabapentin, than pregabalin, but the definitive study in terms of statistical power and duration of follow-up was conducted using pregabalin [65]. Although these agents are similar in terms of mechanism of action, there are differences. Pregabalin appears to have a greater effect in the first hour after surgery than gabapentin, but both drugs have similar effects after that first hour [66]. Adverse events are similar with both agents [63]. Gabapentin is cheaper, which may be an important factor with prolonged therapy [63].

Dexketoprofen-tramadol fixed-dose combination for the optimal prevention of postoperative pain chronification

Multimodal or combination analgesia represents an important advance in the care of postoperative patients in order to reduce pain and avoid persistent painful syndromes. The exact multimodal regimen recommended for postoperative pain control depends on patient factors (age, comorbidities, frailty, drug therapies, substance use disorders, and so on), the type of surgery, and the setting (in hospital or outpatient) [5,47]. While much is known about multimodal pain care, there remain important knowledge gaps.

Interventions to prevent acute postoperative pain from becoming persistent are based on the current understanding of the pain predictors, which may be physiologic, genetic, or psychosocial, or related to the acute postoperative phase [67]. Nerve damage during surgery as well as an inflammatory response to tissue injury during surgery are known contributors to persistent postoperative pain [68]. It is less clear how specific postoperative analgesic regimens may contribute to persistent postoperative pain [69]. A systematic review of 66 trials (n=3,149) identified the main pharmacologic and nonpharmacologic interventions for acute postoperative pain but could not draw conclusions because of the heterogeneity of surgeries and patient populations [67].

When considering individual agents, the dose, time of administration (before, during, or after surgery), route of administration, and type of surgery all may affect results. Furthermore, drug-drug interactions may complicate care and are not always adequately considered by prescribers. For instance, hydrocodone is an opioid in the form of a prodrug that is metabolized via the CYP-2D6 enzyme to its active metabolite; however, the use of an SSRI serves to inhibit the CYP-2D6 substrate and may reduce the effectiveness of hydrocodone [70]. Such considerations are important in patients on polypharmacy, and drug-drug interactions can have serious consequences [71-74].

Clonidine premedication at doses of 4 µg/kg was shown to be effective in reducing postoperative pain in pediatric surgery patients [75]. Although antidepressants are not indicated for the treatment of chronic pain syndromes, venlafaxine may be effective in treating both acute and chronic post-mastectomy pain [76,77]. Lidocaine IV [78], epidural analgesia with a local anesthetic [79], and peripheral nerve blocks [80] have all proven effective in the control of certain types of acute postoperative pain. The IV administration of S-ketamine may be effective when used as part of a multimodal general anesthesia plan in terms of decreasing acute postoperative pain intensity, but its effects last only a short time after surgery [81]. The challenge to clinicians is that the results from these individual studies of specific surgeries in specific populations are not necessarily generalizable to all surgical patients.

Multimodal analgesia has been recognized as an essential component of enhanced recovery after surgery (ERAS) guidance [82]. Multimodal analgesia may prevent chronic postoperative pain [83]. A protocol used at the clinic of one of the authors is based on a stepwise guide to analgesic treatment with fixed-dose dexketoprofen/tramadol (Table 1).

**Table 1:** Clinical protocol from the Surgery Department of the University of Lleida, Spain. Note that for very severe pain, IV dexketoprofen monotherapy is recommended with rescue morphine IV, intravenous

Step	Pain Level on 0-10 Scale	Pain Description	Dexketoprofen/ Tramadol Dose	Rescue
1	1-3	Mild	25 µg/12.5 mg	Acetaminophen/paracetamol
2	4-6	Moderate	25 mg/50 mg	
3	7-8	Severe	25 mg/75 mg	
4	≥ 9	Very severe	IV dexketoprofen 50 mg	Patient-controlled morphine

Dexketoprofen trometamol is a nonselective COX-inhibiting agent available in both parenteral and oral formulations. Dexketoprofen has a rapid onset of action and has proven safe and effective in treating acute pain [84]. When included in a multimodal analgesic regimen, dexketoprofen may exert an opioid-sparing effect [84]. It has a good safety profile and offers the benefit of being an older, established drug with a long history. Its side effects are similar to those of other NSAIDs. As dexketoprofen works well in combination with other agents for multimodal analgesia, it has become available in certain fixed-dose combination formulations with tramadol. It may also be administered as monotherapy or in a “loose dose” combination by simply administering it concurrently with tramadol [4]. Combination therapy with dexketoprofen and tramadol has been evaluated in several clinical trials, which are summarized in Table 2.

**Table 2:** Randomized clinical trials or clinical trials using dexketoprofen/tramadol. Studies were retrieved from PubMed search for “dexketoprofen tramadol” keywords, limited to randomized clinical trials or clinical trials with associated data. The search retrieved 10 articles about studies. Six studies were excluded for various reasons: no evaluation of the drugs used in combination (n=4), animal study (n=1), and no pain-related endpoints (n=1). Studies appear here in alphabetical order based on the surname of the first author [85-88]. Dex, dexketoprofen; Tram, tramadol; NNT, number needed to treat; APAP, acetaminophen/paracetamol

Study	Patient Population	Dose (Dex/Tram)	Results	Safety	Comments
Gay-Escoda et al., 2019 [85]	Single dose, 653 oral surgery patients	25/75 mg	Superior to Tram/APAP and placebo for pain relief; had faster onset of action and longer duration of action	All groups comparable	Comparator Tram/ APAP 75/650 mg
McQuay et al., 2016 [86]	641 total hip arthroplasty patients, five-day study	25/75 mg 25/100 mg; Dex alone Tram alone; rescue metamizole	Combinations provided superior analgesia with best results for 25/100 mg	Comparable among groups	
Moore et al., 2015 [87]	Single dose, 606 oral surgery patients	25/75 mg 12.5/25; Tram monotherapy (37.5 and 75 mg); ibuprofen 400 mg	72% of 25/75 Dex/Tram patients were responders (highest)	Good, comparable among groups	NNT for 25/75 Dex/Tram was 1.6
Moore et al., 2016 [88]	606 patients undergoing abdominal hysterectomy, 3-day study	25/75 mg; Tram 100 mg monotherapy; Dex 25 mg monotherapy	Combination therapy provided superior pain relief	Adverse reactions occurred in <2% of patients except for nausea (4.6%) and vomiting (2.3%)	Combination treatment was safe, effective, and well tolerated

Using a modified Delphi process, expert consensus on the use of dexketoprofen/tramadol combination products for the short-term treatment of acute pain arrived at certain 28 statements, of which 19 achieved expert consensus endorsement [89]. Of those 19 items, seven related specifically to the clinical utilization of dexketoprofen/tramadol for postoperative pain management and are presented in Table 3.

**Table 3:** Expert consensus on the role of dexketoprofen/tramadol fixed-dose combination products for the management of postoperative pain by expert consensus endorsement [89]. D/T FDC, dexketoprofen/tramadol fixed-dose combination product

Statement	Comment
D/T FDC offers effective multimodal analgesia to treat acute postoperative pain in the management of postoperative pain	Multimodal or balanced postoperative analgesic is recommended in guidelines
D/T FDC can provide effective and rapid patient management in day surgery, enabling patients to return to normal activities more quickly	Many procedures are migrating to ambulatory facilities, and outpatient postoperative analgesia is an important consideration
D/T FDC offers effective treatment of moderate-to-severe postoperative pain following major abdominal surgery	For example, in abdominal hysterectomy for benign conditions and other gynecologic surgeries
D/T FDC may allow for early patient mobilization, reduced thromboembolic risk, shorter hospital stay, and better rehabilitation for patients undergoing major orthopedic surgery	Early ambulation and rehabilitation can be crucial following major orthopedic procedures
The use of oral D/T FDC can be envisaged for minor orthopedic surgeries	For example, rotator cuff repair, bunionectomy, and others
D/T FDC is likely to be an effective analgesic following minor surgeries	For example, appendectomies, plastic surgery of soft parts, hernia repair, and others
D/T FDC provides fast, effective relief of postoperative pain following oral and other dental surgeries	Single-dose treatment of dental pain

Multiple medical disciplines can contribute substantially to reducing postoperative pain and the risk that it transitions to persistent postoperative pain. By making smaller incisions and reducing surgical trauma, surgeons play a role. Anesthesiologists using evidence-based multimodal perioperative regimens play a role. Other clinicians should be alert to potential risks for postoperative and persistent postoperative pain and take steps to control for them. Nurses, in particular, should be alert to signs and symptoms of analgesic-related adverse events, as many can be managed without interrupting pain relief. Patients and their families should be advised about what to expect following surgery in terms of pain, healing, and rehabilitation, both to manage expectations and to empower greater adherence and active participation in their recovery.

## Conclusions

Despite increases in the number of surgeries year after year and the migration of more procedures to ambulatory facilities, postoperative pain is still inadequately managed and may contribute to the development of chronic postoperative pain. Chronic pain is costly to both the healthcare system and patients and diminishes the quality of life for the patient. There is an armamentarium of agents, including NSAIDs, acetaminophen, and opioids, that can assist in managing postoperative pain. When administered using multimodal strategies, these can be safe and effective while reducing side effects. In particular, the fixed-dose combination product of dexketoprofen/tramadol can be safe and effective in managing acute postoperative pain.
